# Association of Bioactive Compounds With Antioxidant and Antimicrobial Activities of Allium Extracts Prepared With Commercial Rice Wines and Kaoliang Liquors

**DOI:** 10.1155/jnme/2450595

**Published:** 2026-01-31

**Authors:** Tsan-Chang Chang, Hung-Der Jang

**Affiliations:** ^1^ Department of Nursing, MacKay Junior College of Medicine, Nursing, and Management, Taipei, Taiwan, mkc.edu.tw; ^2^ Department of Food Science, Yuanpei University of Science and Technology, Hsinchu, Taiwan

**Keywords:** *allium*species, antibacterial capacity, antioxidant activity, biological activity, rice wines and kaoliang liquors

## Abstract

**Background:**

*Allium* species are widely used in cooking. They possess antioxidant and antimicrobial activities, which may be due to the sulfur compounds they contain. This study examined the correlation between the active compounds of *Allium* species and their biological activities.

**Methods:**

Extracts from five *Allium* species grown in Taiwan—*A. sativum*, *A. fistulosum*, *A. tuberosum*, *A. fistulosum*, and *A. cepa*—were prepared using commonly available commercially alcohols in Taiwan: rice wine (19RW), front distillation of rice wine (34PRW), 38% kaoliang wine (38KL), and 58% kaoliang wine (58KL). The total phenol content and allicin content of the extracts were measured, and their antioxidant activity and antibacterial activity against common clinical pathogens were evaluated.

**Results:**

The *A. fistulosum* extracts exhibited the highest total phenol content, and the *Allium* extracts derived using 34PRW and 38KL had the highest allicin content. All the *Allium* extracts had favorable antioxidant capacity, with *A. fistulosum*, *A. sativum*, and *A. tuberosum* exhibiting the highest DPPH free radical scavenging rate, TEAC, and SOD antioxidant capacity, respectively. The *A. fistulosum* extracts had a statistically significant increase in the DPPH free radical scavenging rate and total phenol content, the *A. sativum* extracts showed a significant difference in TEAC, and the *A. tuberosum* extracts exhibited a significant difference in SOD antioxidant activity. The sulfur compounds in the *Allium* species were found to be positively correlated with both the species’ antioxidant and antibacterial activities.

**Conclusions:**

These findings demonstrate that the antioxidant and antibacterial activities of *Allium* species are closely linked to their sulfur compounds and that differences among species are reflected in specific antioxidant parameters.

## 1. Introduction

The genus *Allium*, belonging to the family *Amaryllidaceae* and subfamily *Allioideae*, comprises over 900 species [1]. Many *Allium* species, including garlic (*Allium sativum*), shallots (*A. fistulosum*), leeks (*A. tuberosum*), scallions (*A. fistulosum* L. var. *caespitosum*), and onions (*A. cepa*), are perennial plants that grow from bulbs. These plants are commonly used worldwide as vegetables and seasonings, valued for their distinctive flavors primarily due to high levels of organic sulfur compounds such as diallyl disulfide, allicin, and alliin.

Beyond their culinary uses, *Allium* species are recognized for numerous health benefits. Studies have shown that they can lower blood pressure, prevent cardiovascular diseases, combat obesity and tumors, and protect against chronic illnesses [2, 3]. Furthermore, they exhibit strong antioxidant [4–6], antibacterial [7–12], and antifungal [13–16] properties, mainly attributable to their sulfur compounds and polyphenols.

Nutritionally, *Allium* species are rich in amino acids, proteins, sugars, and dietary fibers, and they provide essential vitamins (A, B1, B2, B6, C, E, and nicotinamide) and minerals (sodium, potassium, calcium, magnesium, phosphorus, iron, and zinc) [17, 18]. Notably, they contain S‐allylcysteine, which boosts immune function, and allicin, a compound known for its potent antibacterial action. Allicin acts by reacting with bacterial cystine, forming a crystalline precipitate that disrupts the mercapto groups (–SH) essential for bacterial metabolism and growth, effectively inhibiting bacterial reproduction [7, 8, 19]. Research has demonstrated that garlic and shallot extracts, along with allicin itself, are effective against *Staphylococcus aureus*, *Pseudomonas aeruginosa*, *Candida albicans*, and *Escherichia coli* [11, 20–22]. Moreover, extracts from *A. fistulosum* using commercially available rice wine have shown antifungal activities against pathogens such as *Aspergillus brasiliensis*, *C. albicans*, *Microsporum canis*, *M. gypseum*, *Trichophyton mentagrophytes*, *T. rubrum*, and *T. tonsurans*, with minimum inhibitory concentrations (MICs) ranging from 0.2 to 1.0 mg/mL [14].

Antioxidant studies on different parts of Taiwanese shallots revealed that the leaves of *A. fistulosum* exhibit significantly higher antioxidant activity compared to stems and roots, based on the total phenol content (TPC), DPPH scavenging rate, and overall antioxidant capacity. This contradicts traditional culinary practices, where only the white parts are commonly used and the leaves discarded. Chang et al. [10] specifically recommended the inclusion of *A. fistulosum* leaves in cooking to enhance health benefits. Allicin exhibits antimicrobial activity against *S. aureus*, *Bacillus subtilis*, *E. coli*, and *P. aeruginosa*, with MICs reported in the range of 8–128 μg/mL depending on the strain and study conditions [7, 8, 19]. Taiwanese *A. fistulosum* has also been demonstrated to exhibit outstanding antioxidant and antibacterial effects [11], including activities against clinical pathogens such as *S. aureus*, *B*. *subtilis*, *E. coli*, and *P. aeruginosa*.

Although the antioxidant and antimicrobial properties of *Allium* species have been widely reported [10, 11, 14, 15, 23–27], few studies have focused specifically on high‐quality Taiwanese green onions and garlic. Therefore, this study aimed to evaluate the active compounds (total phenols, allicin, and amino acids) and biological activities (antioxidant and antibacterial) of extracts from different *Allium* species prepared using commercially available local alcohols. In particular, the study sought to develop a rapid method for obtaining active constituents of *A. fistulosum* using rice wine, a common cooking ingredient in Taiwan, enhancing its practical applications in both health promotion and food preservation.

## 2. Materials and Methods

### 2.1. Test Materials and Chemicals

Fresh *A. fistulosum* (scallions) were obtained from local street markets in Sanxing Township; *A. sativum* (organic garlic) and *A. fistulosum* L. var. *caespitosum* (shallots) were purchased from certified agricultural vendors in Yuanlin; *A. cepa* (onions) were acquired from local markets in Hengchun Township; and *A. tuberosum* (leeks) were collected from smallholder farms in Changhua, Taiwan (Figure 1). Five *Allium* species (*A. sativum*, *A. fistulosum*, *A. tuberosum*, *A. fistulosum* var. *caespitosum*, and *A. cepa*) were obtained between June and August 2023. Plant identity was confirmed based on morphological characteristics and cross‐checked with the flora of Taiwan. Fresh samples were transported to the laboratory within 24 h of purchase, washed with distilled water, and stored at 4°C until further processing.

Figure 1
*Allium* species samples used in the experiment. (a) *Allium sativum*, (b) *Allium fistulosum*, (c) *Allium tuberosum*, (d) *Allium fistulosum* L. var. *caespitosum*, and (e) *Allium cepa.*

**Figure:**
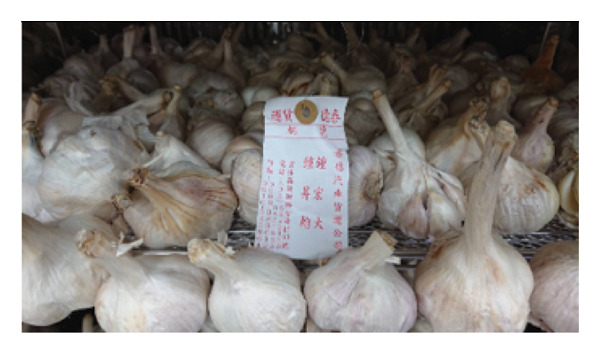
(a)

**Figure:**
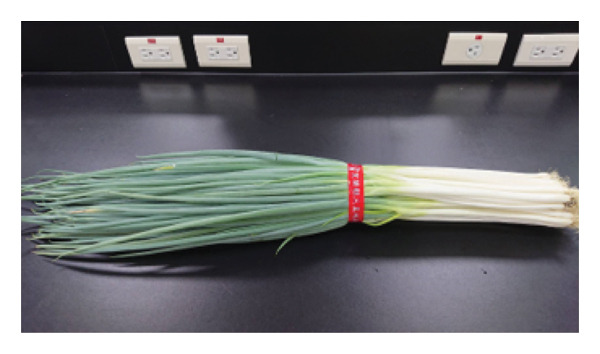
(b)

**Figure:**
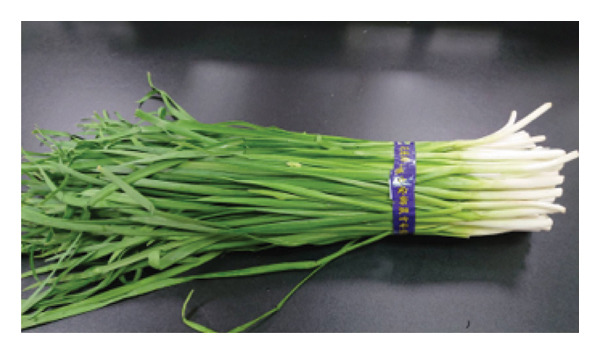
(c)

**Figure:**
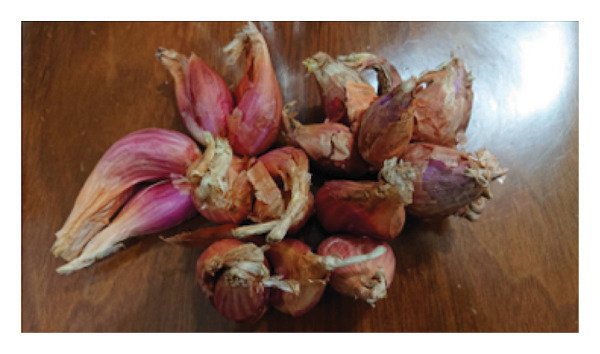
(d)

**Figure:**
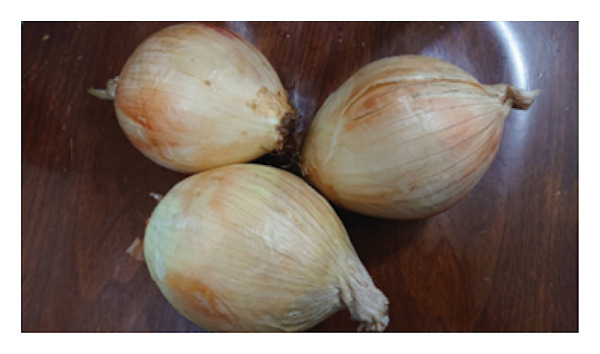
(e)

To assess the biological activities of Allium extracts, various local alcohols produced by the Taiwan Tobacco and Liquor Corporation were purchased: rice wine (19RW, 19.5% ABV), front distillation of pure rice wine (34PRW, 34% ABV), and kaoliang liquors (38KL, 38% ABV; 58KL, 58% ABV). In this study, rice wine (19RW), front distillation of rice wine (34PRW), and kaoliang liquors (38KL, 58KL) were used as extraction solvents. Hereafter, they are collectively referred to as “rice wines and kaoliang liquors.” To evaluate the influence of solvent polarity on the extraction of phenolic compounds and allicin, four ethanol–water mixtures (19RW, 34PRW, 38KL, and 58KL) were selected as extraction solvents. These formulations represent a controlled gradient of polarity achieved by adjusting the ethanol concentration, as the dielectric constant of ethanol–water mixtures decreases with increasing ethanol content. Solvent systems with lower ethanol levels exhibit higher polarity and stronger hydrogen‐bonding interactions, whereas higher ethanol concentrations provide enhanced organic solvency and improved permeability through plant tissues. This range (19%–58% v/w) was chosen to cover polarity values known to affect the solubility and diffusion of semipolar phytochemicals, enabling the systematic comparison of extraction performance across the solvent gradient. Extraction using these mixtures was carried out under identical operational conditions as described below.

Standard allicin products and bacterial culture media, including nutrient agar (NA), nutrient broth, and Mueller–Hinton agar, were sourced from Difco Chemical Co. (USA). Bacterial strains (*Acinetobacter baumannii*, *B. subtilis*, *E. coli*, *Klebsiella pneumoniae*, *P. aeruginosa*, and *S. aureus*) were obtained from the Bioresource Collection and Research Center of the Food Industry Research Institute (Hsinchu, Taiwan).

Analytical reagents such as DPPH, ABTS, Trolox, Folin–Ciocalteu reagent, quercetin, and (+)‐catechin were purchased from Sigma Chemical Co. (USA), and potassium persulfate was purchased from Merck Co. (USA).

### 2.2. Optimal Extraction Solvent for Allium Species

*Allium* materials were vacuum dried at 50°C, chopped, and mixed with each solvent at a 1:2 (w/v) ratio. After storage in 500‐mL serum bottles for 2 weeks, the mixtures were filtered to obtain clear extracts, which were then stored at 4°C for further testing.

### 2.3. Active Compounds in Allium Extracts

#### 2.3.1. TPC

TPC was determined using the Folin–Ciocalteu method [28] following protocols used in scallion extract studies [10, 11]. Briefly, 1.0 mL of Folin–Ciocalteu reagent was mixed with extract, followed by the addition of sodium carbonate. After incubation at room temperature, the solution was centrifuged at 12, 000 × *g*, and absorbance was measured at 730 nm using a UV–visible spectrophotometer. A calibration curve with gallic acid was used to express the results as mg gallic acid equivalents (GAEs) per gram of sample (mg GAE/g). All measurements were performed in triplicate.

#### 2.3.2. Allicin Content

Allicin content was analyzed by high‐performance liquid chromatography (HPLC) using an Agilent 1100 system with a UV–visible diode array detector and a Finnigan LCQ‐DECA mass spectrometer. Separation was achieved on a Phenomenex Luna C18(2) column (150 × 4.6 mm, 5 μm). The mobile phase included Solvent A (0.1% trifluoroacetic acid in Milli‐Q water) and Solvent B (acetonitrile), with a gradient from 10% to 95% B over 15 min. The flow rate was 1.0 mL/min at 30°C. Allicin solutions were diluted in methanol, and 1 μL was injected. Detection was conducted at 250 ± 40 nm, with allicin eluting at 9.95 min.

Extracts were first filtered through Whatman No. 1 filter paper, followed by 0.45‐μm PTFE membrane filtration under vacuum. For HPLC analysis, all samples were passed through 0.22‐μm syringe filters to remove particulate matter and ensure chromatographic accuracy. HPLC was used to measure the allicin content, establishing a strong linear calibration curve (*Y* = 29.05*X* − 1.72, *r* ^2^ = 0.999). This method aligned with Fujisawa et al. [29], who confirmed HPLC’s effectiveness for allicin detection at 220 nm.

#### 2.3.3. Free Amino Acid Content

Free amino acids were quantified by the Food Industry Research and Development Institute (FIRDI) using ion‐exchange chromatography, based on CNS 12632 [30] guidelines from the Bureau of Standards, Metrology and Inspection. The method, adapted from Moore et al. [31], is standardized for determining amino acids in fruit and vegetable products. FIRDI also provided data on antioxidant and antibacterial activity. Amino acid data were obtained from FIRDI. These results are based on single measurements without replication; therefore, standard deviations and post hoc statistical comparisons could not be calculated.

The free amino acid content was determined using the ninhydrin colorimetric method with slight modifications. Briefly, 1 mL of extract was mixed with 1 mL of 2% ninhydrin reagent and incubated in a boiling water bath for 15 min. After cooling to room temperature, absorbance was measured at 570 nm using a spectrophotometer (Hitachi U‐2900, Japan). L‐leucine was used as the standard, and the calibration curve was established over the range of 0–200 μg/mL (*r* ^2^ = 0.998). The results were expressed as mg leucine equivalents per g dry weight.

### 2.4. Antioxidant Capacity Assays of Allium Extracts

#### 2.4.1. DPPH Scavenging Rate

The DPPH free radical scavenging activity was assessed following Gyamfi et al. [32] and prior *A. fistulosum* research [8, 9]. A 100‐μL sample of extract or catechin standard (5 μg/mL) was mixed with 100 μL of 0.2 mM DPPH in ethanol. The mixture was shaken and kept in the dark for 30 min at room temperature. Absorbance was read at 517 nm. The DPPH scavenging rate was calculated as follows: [1 − (sample absorbance/control group absorbance)] × 100. IC_50_ values, indicating the concentration required to scavenge 50% of DPPH radicals, were determined to compare antioxidant strength. Experiments were repeated three times.

#### 2.4.2. Trolox Equivalent Antioxidant Capacity (TEAC)

TEAC was measured according to Re et al. [33] by reacting ABTS+ with sample extracts. ABTS+ was generated and diluted to an absorbance of 0.7 ± 0.02 at 734 nm. The reaction with samples resulted in a decrease in absorbance, which was measured spectrophotometrically. A standard curve using Trolox was used to calculate antioxidant capacity, expressed as Trolox equivalents.

#### 2.4.3. Superoxide Dismutase (SOD) Activity

SOD activity was measured using the nitroblue tetrazolium (NBT) reduction method. The reaction mixture contained 50 mM phosphate buffer (pH 7.8), 130 mM methionine, 0.75 mM NBT, 0.1 mM EDTA, and 20 μM riboflavin. The reaction was initiated by placing the samples under fluorescent light (4000 lux) for 15 min at 25°C. Absorbance was measured at 560 nm using a microplate reader (BioTek Synergy HTX, USA). One unit of SOD activity was defined as the amount of enzyme required to inhibit 50% of NBT photoreduction. A standard curve was prepared using purified SOD (Sigma, USA), with concentrations ranging from 0 to 500 U/mL (*r* ^2^ = 0.995). The results were expressed as U/mg protein.

#### 2.4.4. Calibration Curves and Instrumentation

Antioxidant activities were assessed using the DPPH, TEAC, and SOD assays. For each method, standard curves were generated based on the known concentrations of reference compounds, and the correlation coefficients (*r* ^2^) were used to validate the linearity of the responses.

• DPPH Radical Scavenging Assay: The calibration curve was established using ascorbic acid as the standard, yielding the linear equation *Y* = 0.996*X* − 0.275 with an *r* ^2^ = 0.998. Absorbance was measured at 517 nm using a UV–Vis spectrophotometer (Shimadzu UV‐1800, Kyoto, Japan).
• TEAC Assay: Trolox was used as the reference antioxidant, and the calibration curve followed the equation *Y* = 0.993*X* − 0.689, with a correlation coefficient of *r* ^2^ = 0.993. Absorbance was recorded at 734 nm using the same spectrophotometric system.
• SOD Activity Assay: Standard SOD solutions were used to construct the calibration curve with the equation *Y* = 4.991*X* − 2.887 and *r* ^2^ = 0.997. Absorbance readings were taken at 560 nm using a microplate reader (BioTek Synergy HT, Winooski, VT, USA).

Standard concentrations were prepared by serial dilution to cover the linear detection range for each assay. All measurements were performed in triplicate to ensure accuracy and reproducibility.

#### 2.4.5. Extraction Procedure

Plant samples were subjected to an optimized extraction protocol to obtain antioxidant‐rich extracts while minimizing the degradation of bioactive compounds.

• Solvent: A 70% (v/v) ethanol solution was selected based on its polarity and efficiency in extracting phenolic and flavonoid compounds.
• Temperature Control: Extractions were carried out at a controlled temperature of 25°C ± 2°C to preserve thermolabile antioxidant compounds.
• Agitation: Samples were continuously agitated at 150 rpm using an orbital shaker (IKA KS 4000 i Control, Staufen, Germany) for 24 h to ensure efficient solute–solvent interaction.
• Light Protection: To prevent photo‐oxidation, all extraction steps were conducted in amber glass containers and under low‐light conditions.
• Extraction Yield: After extraction, the solvent was removed under reduced pressure using a rotary evaporator (Büchi R‐210, Flawil, Switzerland). The resulting crude extract was weighed, and the extraction yield was calculated as a percentage of the dry plant material weight.

This protocol ensured high recovery of antioxidant constituents while maintaining their chemical stability and biological activity.

### 2.5. Antibacterial Capacity of Allium Extracts

Antibacterial activity was tested using the disk agar diffusion and tube dilution methods, following protocols by Fujisawa et al. [29] and Mnayer et al. [34]. Six bacterial strains were tested: *A*. *baumannii*, *B*. *subtilis*, *E*. *coli*, *K*. *pneumoniae*, *P*. *aeruginosa*, and *S*. *aureus*. In the disk diffusion method, agar plates inoculated with bacterial cultures were incubated at 37°C for 24 h, and inhibition zone diameters were measured in millimeters. In the tube dilution test, serially diluted extracts were added to bacterial suspensions (∼10^5^ CFU/mL) and incubated at 37°C for 24 h. Bacterial growth was assessed using a photoelectrometer at 600 nm to determine the MIC. Penicillin (10 μg/mL) and tetracycline (30 μg/mL) were used as positive controls.

### 2.6. Statistical Analysis

All experiments were conducted in triplicate. The results are presented as mean ± standard deviation. Statistical significance was evaluated using one‐way analysis of variance (ANOVA) followed by Duncan’s multiple range test at a 95% confidence level (*p* < 0.05), using SPSS 14 (SPSS Inc., Chicago, USA).

## 3. Results and Discussion

### 3.1. Active Compounds in Allium Extracts

#### 3.1.1. TPC

Table 1 presents the TPC of *Allium* extracts derived using various commercially available alcoholic beverages as solvents. The *A. fistulosum* extract obtained with 34 MTW had the highest TPC (88.72 mg GAE/g), followed by the extract using 38 KL (78.71 mg GAE/g). These levels significantly exceeded those of other *Allium* species. *A. sativum* extracts showed 55.87 and 55.11 mg GAE/g when using 34 PRW and 38 KL, respectively, while *A. cepa* extracts exhibited 40.84 and 39.50 mg GAE/g. In contrast, *A. tuberosum* and *A. fistulosum L. var. caespitosum* extracts had much lower phenol contents (6.10 and 4.37 mg GAE/g, respectively).

**Table 1:** Total phenol and allicin content of *Allium* extracts obtained using alcohols.

Material	Extract solvent	Total phenol content (mg GAE^∗^/g)	Allicin content (μL/mL)
*Allium sativum*	19RW	43.54 ± 0.87^ef∗∗^	52.03 ± 1.35^c^
	34PRW	**55.87 ± 0.16** ^**d**^	**95.85 ± 2.53** ^**a**^
	38KL	55.11 ± 0.53^d^	94.53 ± 2.44^a^
	58KL	46.21 ± 1.15^e^	32.60 ± 2.10^f^

*Allium fistulosum*	19RW	66.87 ± 2.40^c^	49.53 ± 1.14^cd^
	34PRW	**81.72 ± 5.96** ^**a**^	**93.40 ± 1.75** ^**ab**^
	38KL	78.71 ± 3.25^a^	91.13 ± 0.35^b^
	58KL	71.43 ± 2.57^b^	28.43 ± 1.66^g^

*Allium tuberosum*	19RW	5.71 ± 1.00^i^	16.27 ± 0.81^j^
	34PRW	**6.10 ± 0.95** ^**i**^	**21.80 ± 1.91** ^**h**^
	38KL	5.80 ± 0.84^i^	19.27 ± 2.15^hi^
	58KL	5.46 ± 1.44^i^	15.67 ± 1.86^j^

*Allium fistulosum* L. var. caespitosum	19RW	2.64 ± 0.43^i^	17.57 ± 1.37^ij^
	34PRW	**4.37 ± 0.23** ^**i**^	**28.50 ± 0.98** ^**g**^
	38KL	3.98 ± 0.22^i^	25.73 ± 1.01^g^
	58KL	2.49 ± 0.56^i^	16.47 ± 0.61^j^

*Allium cepa*	19RW	28.82 ± 1.88^h^	37.63 ± 1.32^c^
	34PRW	39.50 ± 4.06^g^	48.40 ± 1.25^d^
	38KL	**40.84 ± 3.57** ^fg^	**52.23 ± 1.27** ^c^
	58KL	30.38 ± 1.94^h^	39.27 ± 2.00^e^

*Note:* Bold values indicate the highest content.

^∗^GAE, gallic acid equivalent.

^∗∗^Within columns, data followed by the different lower‐case letter are significantly different at *p* < 0.05, as determined using Duncan’s multiple range test.

Previous studies have reported that aqueous and water–ethanol mixtures are effective solvents for extracting phenolic compounds from *Allium* species. For example, Nuutila et al. [35] found that aqueous ethanol (50%) extracts of onion contained 32.4–45.8 mg GAE/g DW, while pure water extracts yielded 18.6–24.3 mg GAE/g DW. Similarly, Kim et al. [36] reported that garlic extracted with 50% ethanol exhibited a TPC of 52.1 mg GAE/g DW, compared to 28.9 mg GAE/g DW for water extracts. These values are within the range of our results using rice wine and kaoliang liquors (e.g., *A. fistulosum* 34PRW, 88.72 mg GAE/g; *A. sativum* 34PRW, 55.87 mg GAE/g), highlighting that rice wines—being aqueous ethanol mixtures—provide comparable or even higher extraction efficiency than conventional hydroalcoholic solvents.

The TPC of *Allium* plants including garlic, onion, and scallion, has been reported in several studies. A complex mixture of phenolic compounds was identified in the phenolic components of *Allium* extracts. According to the literature [25, 26, 37], the most commonly identified phenolic compounds in *Allium* species include quercetin, rutin, caffeic acid, ferulic acid, chlorogenic acid, and p‐coumaric acid. The composition and relative abundance of these compounds vary by species. For example, Barbu et al. [25, 26] reported that garlic contains a TPC of 278 μg GAE/g, with *p*‐coumaric acid comprising 102 μg/g. In contrast, scallion was found to have a higher TPC of 327 μg GAE/g, with ferulic acid and p‐coumaric acid present at 499 and 302 μg/g, respectively. Similarly, Kim et al. [37] reported a TPC of 278 μg GAE/g in garlic, with chlorogenic acid and p‐coumaric acid measured at 36 and 26 μg/g, respectively. We did not perform in‐depth compositional analysis of phenolic profiles. Instead, Table 2 summarizes the representative values from previous studies to provide context for the contribution of individual phenolics to overall TPC. Khalili et al. [38] found that ethyl acetate yielded the highest phenol content (423.95 ± 11.39 mg GAE/g) in red onion skin extracts. However, they noted that highly polar solvents like ethyl acetate and n‐butanol led to lower total phenol recovery rates. Lisanti et al. [39] reported a TPC of 6.61 ± 0.87 mg GAE/g in Italian onions (*A. cepa*), significantly lower than the 81.72 mg GAE/g achieved for *A. fistulosum* in the present study using 34 PRW.

**Table 2:** Commonly detected phenolic compounds in *Allium* species and their approximate contributions to TPC.

Compounds	Approximate percentage of TPC^∗^ (%)	Notes
Quercetin	30–50	Higher content typically found in onions
Rutin	10–20	A derivative of quercetin
Caffeic acid	5–10	Commonly found in garlic and scallions
Ferulic acid	5–10	Commonly found in garlic and scallions
Chlorogenic acid	3–8	Some onion varieties exhibit a higher content
p‐Coumaric acid	1–5	Considered a minor phenolic acid

*Note:* Data are compiled from literature reports [30, 31], not experimental values obtained in this study.

^∗^TPC, total phenolic content.

#### 3.1.2. Allicin Content

Table 1 shows that *A. sativum* extracts exhibited the highest allicin contents, 95.85 and 94.53 μL/mL, when extracted with 34 PRW and 38 KL, respectively. *A. fistulosum* extracts followed closely with 93.40 and 91.13 μL/mL. The allicin content was lower in *A. cepa* extracts (52.23 and 48.40 μL/mL) and lowest in *A. tuberosum* and *A. fistulosum L. var. caespitosum* (21.80 and 28.50 μL/mL, respectively).

These findings highlight that 34 PRW and 38 KL are effective solvents for extracting allicin. Previous research by Țigu et al. [40] found allicin concentrations of 380 and 20 μg/mL in *A. sativum* and *A. fistulosum*, respectively, while Sayadi et al. [41] reported a significant variation in the allicin content among *Allium* species and plant tissues, with *A. sativum* bulbs showing the highest concentration (1.185%). The present study’s higher allicin yields further emphasize the influence of solvent choice. Accordingly, the results of this study, in line with numerous previous reports [7, 8, 19], demonstrate that allicin indeed possesses antibacterial activity and is capable of penetrating bacterial cell membranes to exert its antimicrobial effects.

#### 3.1.3. Amino Acid Content

Table 3 summarizes the free amino acid contents of *Allium* species extracts using commercially available front‐distillation rice wine. *A. sativum* exhibited the highest total amino acid content (1843.12 mg/100 g), followed by *A. fistulosum* (492.68 mg/100 g), *A. tuberosum* (81.97 mg/100 g), *A. fistulosum L. var. caespitosum* (87.42 mg/100 g), and *A. cepa* (48.36 mg/100 g). In *A. sativum*, L‐arginine was predominant (1036.63 mg/100 g), followed by asparagine (296.47 mg/100 g) and lysine (78.57 mg/100 g). In *A. fistulosum*, arginine (116.55 mg/100 g) and alanine (75.79 mg/100 g) were most abundant. Aspartic acid and alanine were predominant in *A. tuberosum* and *A. cepa*.

**Table 3:** Content of 20 common amino acids.

Amino acid	*Allium sativum* (mg/100 g)	*Allium fistulosum* (mg/100 g)	*Allium tuberosum* (mg/100 g)	*Allium fistulosum* L. var. *caespitosum* (mg/100 g)	*Allium cepa* (mg/100 g)
Alanine	65.35^∗^	75.79	19.83	42.03	12.94
Arginine	1036.63	116.55	12.69	18.94	1.21
Asparagine	296.47	18.78	—	—	—
Aspartic acid	15.57	21.58	22.25	2.68	16.39
Cysteine	—	—	—	6.02	—
Glutamic acid	52.92	8.6	18.36	2.02	11.92
Glutamine	—	—	—	—	—
Glycine	2.97	11.29	2.47	2.83	1.46
Histidine	14.21	1.10	—	—	—
Isoleucine	17.05	13.64	0.32	0.81	0.20
Leucine	34.73	25.95	0.36	0.40	0.32
Lysine	78.57	19.19	0.89	0.81	—
Methionine	7.68	2.04	0.19	0.25	—
Phenylalanine	18.84	15.67	—	—	—
Proline	31.51	42.90	—	—	—
Serine	55.75	24.82	0.51	0.25	0.57
Threonine	21.08	15.06	0.70	0.30	0.53
Tryptophan	15.96	—	—	—	—
Tyrosine	46.51	30.13	—	—	0.56
Valine	31.32	49.59	3.40	10.08	2.26
Total amino acid	**1843.12**	**492.68**	**81.97**	**87.42**	**48.36**

*Note:* Bold values indicate the total amino acid content.

^∗^Values represent single determinations obtained from the Food Industry Research and Development Institute (FIRDI), Taiwan. No replicates were available; therefore, statistical parameters (standard deviation, post hoc tests) could not be performed. “—” indicates content not detected.

Bhat [42] previously reported the presence of organosulfur compounds, thiosulfinates, and various amino acids across *Allium* species. This study confirms considerable variability depending on the species and extraction method. Although the correlation between TPC and amino acid contents was not directly examined here, Kovačević et al. [43] found positive correlations between the TPC and amino acids such as threonine, methionine, and lysine in *A. ursinum*. In the present study, *A. sativum* extracts also contained notable amounts of these amino acids, suggesting potential links between phenolic and amino acid profiles.

### 3.2. Antioxidant Capacity of Allium Extracts

#### 3.2.1. DPPH Scavenging Rate

Table 4 shows the DPPH scavenging rates and IC_50_ values of *Allium* extracts. *A. fistulosum* extracts using 38KL and 34PRW exhibited the highest scavenging rates (68.50% and 68.47%, IC_50_ = 5.97 and 6.03 μg/mL, respectively). These were followed by *A. tuberosum* (54.33%, IC_50_ = 8.27 μg/mL), *A. sativum* (48.57%, IC_50_ = 6.47 μg/mL), *A. cepa* (48.63%, IC_50_ = 7.20 μg/mL), and *A. fistulosum L. var. caespitosum* (36.27%, IC_50_ = 12.47 μg/mL) extracted using 34PRW. Overall, *A. fistulosum* extracts showed the highest antioxidant activity among all tested species.

**Table 4:** DPPH free radical scavenging rates and IC_50_ and TEAC values.

Material	Extract solvent	DPPH free radical scavenging rate (%)	IC_50_ (μg/mL)	TEAC (μmol/L)
Allium sativum	19RW	45.63 ± 1.60^∗de^	6.80 ± 0.10^efg^	9.43 ± 2.90^cde^
	34PRW	**48.57 ± 1.30** ^d^	**6.47 ± 0.06** ^fg^	**16.30 ± 1.90** ^a^
	38KL	48.07 ± 1.27^de^	6.53 ± 0.06^fg^	15.73 ± 2.00^a^
	58KL	43.63 ± 1.16^e^	6.83 ± 0.06^efg^	10.63 ± 2.67^cde^

Allium fistulosum	19RW	59.33 ± 1.63^b^	6.57 ± 0.12^efg^	5.67 ± 0.06^hi^
	34PRW	68.47 ± 1.59^a^	6.03 ± 0.06^g^	**7.60 ± 1.10** ^efgh^
	38KL	**68.50 ± 0.87** ^a^	**5.97 ± 0.06** ^g^	7.33 ± 0.49^fgh^
	58KL	54.43 ± 3.25^c^	6.50 ± 0.10^fg^	6.43 ± 0.76^ghi^

Allium tuberosum	19RW	38.67 ± 1.60^f^	10.57 ± 0.93^bc^	8.27 ± 0.40^defgh^
	34PRW	**54.33 ± 0.61** ^c^	**8.27 ± 0.57** ^defg^	**10.87 ± 0.06** ^cd^
	38KL	53.87 ± 0.31^c^	8.80 ± 1.01^cde^	10.20 ± 0.30^cde^
	58KL	42.60 ± 1.11^e^	9.97 ± 0.55^cd^	8.30 ± 1.03^defgh^

Allium fistulosum L. var. caespitosum	19RW	26.60 ± 2.00^g^	14.17 ± 2.81^a^	3.73 ± 0.15^i^
	34PRW	**36.27 ± 5.46** ^f^	**12.47 ± 1.85** ^ab^	5.57 ± 0.74^hi^
	38KL	34.53 ± 4.64^f^	12.83 ± 1.70^a^	**5.50 ± 0.82** ^hi^
	58KL	25.77 ± 2.91^g^	14.40 ± 2.11^a^	14.63 ± 2.25^ab^

*Allium cepa*	19RW	26.77 ± 1.34^g^	10.70 ± 2.10^bc^	8.03 ± 1.65^defgh^
	34PRW	**48.63 ± 3.25** ^d^	**7.20 ± 0.40** ^efg^	**11.97 ± 3.46** ^bc^
	38KL	44.03 ± 4.41^de^	8.57 ± 1.34^cdef^	11.37 ± 2.97^c^
	58KL	28.93 ± 2.60^g^	9.77 ± 1.30^cd^	7.67 ± 0.85^efgh^

Abbreviation: TEAC, Trolox equivalent antioxidant capacity.

^∗^Within columns, data followed by the different lower‐case letter are significantly different at *p* < 0.05, as determined using Duncan’s multiple range test.

^∗∗^A concentration of 5 μg/mL of catechin, exhibiting a DPPH scavenging rate of 75.38% ± 0.14%, was used as the control group. IC_50_ represents the concentration required to inhibit 50% of DPPH free radicals.

Supporting literature by Khalili et al. [38] found a strong correlation between DPPH radical scavenging activity and total flavonoid content in red onion peel extracts. Lisanti et al. [39] also reported a significant antioxidant activity (22.90 ± 0.01 mmol Trolox equivalents/g) in *A. cepa* extracts. In this study, *A. fistulosum* extracted with 38KL displayed the highest DPPH scavenging rate (68.50%).

#### 3.2.2. TEAC

As shown in Table 4, TEAC values were the highest in the *A. sativum* extract derived using 34PRW (16.30 μmol/L), followed by *A. cepa* (11.97 μmol/L), *A. tuberosum* (10.87 μmol/L), *A. fistulosum* (7.60 μmol/L), and *A. fistulosum L. var. caespitosum* (5.50 μmol/L with 38KL). Thus, the *A. sativum* extract demonstrated the strongest antioxidant capacity based on TEAC values.

Lisanti et al. [39] reported TEAC/ABTS values of 21.31 ± 0.41 mmol Trolox equivalents/g dry weight for onion extracts. Although slightly lower in this study, the 16.30 μmol/L value for *A. sativum* suggests good antioxidant potential when extracted with 34PRW.

#### 3.2.3. SOD Antioxidant Capacity

Table 5 presents the SOD activities for *Allium* extracts. *A. tuberosum* extracts derived with 34PRW and 38KL showed the highest SOD capacities (412.17 and 411.10 U/mg protein), closely followed by *A. fistulosum* (411.83 and 407.63 U/mg protein). *A. sativum* (335.03 U/mg), *A. cepa* (275.23 U/mg), and *A. fistulosum L. var. caespitosum* (243.20 U/mg) had lower activities.

**Table 5:** SOD antioxidant activity.

Materials	Extract solvent	SOD^∗^ antioxidant capacity (U/mg protein)
Allium sativum	19RW	286.10 ± 17.62^∗∗de^
	34PRW	**335.03 ± 6.96** ^c^
	38KL	326.40 ± 12.10^c^
	58KL	303.17 ± 12.75^d^

Allium fistulosum	19RW	365.07 ± 13.20^b^
	34PRW	**411.83 ± 10.85** ^a^
	38KL	407.63 ± 9.85^a^
	58KL	375.23 ± 8.45^b^

Allium tuberosum	19RW	372.10 ± 11.86^b^
	34PRW	**412.17 ± 8.58** ^a^
	38KL	411.10 ± 2.40^a^
	58KL	395.37 ± 15.24^a^

Allium fistulosum L. var. caespitosum	19RW	196.43 ± 9.47^h^
	34PRW	**243.20 ± 11.38** ^fg^
	38KL	240.27 ± 9.83^fg^
	58KL	204.53 ± 6.63^h^

*Allium cepa*	19RW	187.27 ± 9.01^h^
	34PRW	257.07 ± 6.37^f^
	38KL	**275.23 ± 9.65** ^e^
	58KL	234.20 ± 10.02^g^

^∗^SOD, superoxide dismutase.

^∗∗^Within columns, data followed by the different lower‐case letter are significantly different at *p* < 0.05, as determined using Duncan’s multiple range test.

Kurnia et al. [44] identified quercetin, kaempferol, rutin, caffeic acid, ferulic acid, and chlorogenic acid as the major phenolic compounds in *Allium* extracts. These compounds have been reported to modulate antioxidant enzymes, particularly SOD, through different mechanisms. Quercetin and kaempferol can enhance SOD activity by upregulating gene expression via the Nrf2 pathway, while rutin has been shown to stabilize cellular redox balance and indirectly increase SOD activity. Hydroxycinnamic acids such as caffeic, ferulic, and chlorogenic acid contribute to scavenging reactive oxygen species, thereby reducing oxidative burden and sustaining SOD activity. In line with these reports, the elevated SOD activity observed in *A. tuberosum* extracts in our study may be attributed to the abundance of these specific compounds.

### 3.3. Antibacterial Activity of Allium Extracts

The antibacterial activities of *Allium* extracts were assessed using the disk agar diffusion method (Table 6). *A. sativum* extracts consistently showed the strongest antibacterial activity across multiple strains, regardless of the solvent used (19RW, 34PRW, 38KL). It should be noted that antibiotics were included only as positive controls and not incorporated into Duncan’s post hoc test. As a result, direct statistical comparisons between antibiotics and *Allium* extracts were not performed, which represents a limitation of the current analysis.

**Table 6:** Antibacterial activity against common clinical pathogens.

Material	Ab‐19606	Bs‐11774	Ec‐25922	Kp‐9633	Pa‐19429	Sa‐6538
19RW *Allium sativum*	21.97 ± 0.25^b^	10.00 ± 0.00^e^	14.77 ± 0.25^c^	13.90 ± 0.56^b^	9.00 ± 0.00^gh^	20.77 ± 0.74^b^
34PRW *Allium sativum*	**25.37 ± 0.65** ^**a**^	11.03 ± 0.21^d^	**17.77 ± 0.25** ^**a**^	15.67 ± 0.29^a^	9.33 ± 0.29^gh^	**31.33 ± 0.76** ^**a**^
38KL *Allium sativum*	24.73 ± 0.65^a^	12.00 ± 0.44^c^	15.50 ± 0.50^b^	**15.83 ± 0.29** ^**a**^	9.17 ± 0.29^gh^	23.00 ± 1.00^ab^
19RW *Allium fistolosum*	9.27 ± 0.15^h^	16.33 ± 0.76^b^	8.67 ± 0.29^gh^	8.27 ± 0.25^e^	10.17 ± 0.35^ef^	12.33 ± 0.29^d^
34PRW *Allium fistolosum*	9.27 ± 0.06^h^	**17.67 ± 0.29** ^**a**^	10.20 ± 0.61^e^	9.00 ± 0.50^d^	**13.10 ± 0.85** ^**a**^	13.00 ± 0.50^cd^
38KL *Allium fistolosum*	11.20 ± 0.10^f^	17.27 ± 0.40^a^	10.10 ± 0.26^e^	9.17 ± 0.29^d^	12.27 ± 0.25^b^	12.77 ± 0.25^cd^
19RW *Allium tuberosum*	13.30 ± 0.89^e^	—, —, —	—, —, —	13.17 ± 0.29^c^	10.47 ± 0.61^def^	19.17 ± 0.58^bc^
34PRW *Allium tuberosum*	14.00 ± 0.50^d^	8.67 ± 0.29^f^	8.83 ± 0.29^fg^	13.83 ± 0.29^b^	11.17 ± 0.29^cd^	20.10 ± 0.36^b^
38KL *Allium tuberosum*	16.27 ± 0.52^c^	9.03 ± 0.11^f^	9.33 ± 0.29^f^	14.17 ± 0.58^b^	10.83 ± 0.29^de^	20.00 ± 0.50^b^
19RW *Allium fistulosum* L. var. *caespitosum*	—, —, —	—, —, —	—, —, —	—, —, —	8.83 ± 0.29^h^	—, —, —
34PRW *Allium fistulosum* L. var. *caespitosum*	—, —, —	—, —, —	8.17 ± 0.29^h^	—, —, —	9.33 ± 0.29^gh^	—, —, —
38KL *Allium fistulosum* L. var. *caespitosum*	10.10 ± 0.36^g^	—, —, —	8.33 ± 0.29^gh^	—, —, —	9.40 ± 0.17^gh^	—, —, —
19RW *Allium cepa*	—, —, —	—, —, —	11.67 ± 0.29^d^	—, —, —	9.77 ± 0.25^fg^	—, —, —
34PRW *Allium cepa*	—, —, —	—, —, —	12.17 ± 0.29^d^	—, —, —	12.10 ± 0.36^b^	—, —, —
38KL *Allium cepa*	—, —, —	—, —, —	11.83 ± 0.29^d^	—, —, —	11.83 ± 0.76^bc^	—, —, —
19RW	—, —, —	—, —, —	—, —, —	—, —, —	—, —, —	—, —, —
34PRW	—, —, —	—, —, —	—, —, —	—, —, —	—, —, —	—, —, —
38KL	—, —, —	—, —, —	—, —, —	—, —, —	—, —, —	—, —, —
Penicillin	10.67 ± 0.29	27.07 ± 1.40	10.60 ± 0.36	9.33 ± 0.29	11.40 ± 0.20	24.97 ± 0.55
Tetracycline	14.63 ± 0.32	22.33 ± 2.25	26.53 ± 2.41	12.67 ± 0.29	18.33 ± 2.25	12.17 ± 0.76

*Note:* Antibacterial activity of *Allium* extracts (zone of inhibition, mm) against clinical strains. Values are expressed as mean ± SD (*n* = 3). Different superscript letters within the same column indicate significant differences among *Allium* extracts (*p* < 0.05, Duncan’s multiple range test). Antibiotic controls of penicillin (10 μg/mL) and tetracycline (30 μg/mL) were included as positive references but were not subjected to Duncan’s test; therefore, no statistical comparisons were made between plant extracts and antibiotics. “—” indicates no antibacterial effect observed. The strains used in this study were *Ac. baumannii* (Ab‐19606), *B. subtilis* (Bs‐11774), *E. coli* (Ec‐25922), *K. pneumoniae* (Kp‐9633), *P. aeruginosa* (Pa‐19429), and *S. aureus* (Sa‐6538). Bold values indicate the highest zone of inhibition (mm).

The *A. sativum* extracts derived with 34PRW and 38KL produced inhibition zones of 25.37 and 24.73 mm against *B. subtilis*, respectively—the largest zones observed. For *B. cereus*, *A. fistulosum* extracts using 34PRW and 38KL (17.67 and 17.27 mm) demonstrated optimal activity. Against *E. coli*, the *A. sativum* extract (34PRW) had the highest inhibition zone (17.77 mm). Similarly, for *K. pneumoniae*, *A. sativum* extracts showed inhibition zones of 25.37 and 24.73 mm. *A. tuberosum* extracts derived using 38KL and 34PRW showed moderate activity against *K. pneumoniae* (14.17 and 13.83 mm), and the *A. fistulosum* 34PRW extract exhibited a notable activity against *P. aeruginosa* (13.10 mm). Against *S. aureus*, the *A. fistulosum* 34PRW extract produced the largest inhibition zone (31.33 mm). In contrast, *A. fistulosum L. var. caespitosum* and *A. cepa* extracts showed no antibacterial activity against *B. cereus*, *K. pneumoniae*, or *S. aureus*. The alcohol solvents alone (19RW, 34PRW, 38KL) showed no antibacterial effect. The results were compared to standard controls (penicillin 10 μg/mL and tetracycline 30 μg/mL).

Khalili et al. [38] also observed that red onion peel extracts exhibited a stronger antibacterial activity against *S. aureus* than against *E. coli* and *Salmonella typhimurium*. The present findings confirm that *Allium* extracts prepared using 34PRW and 38KL are effective against common clinical strains. Consistent with a wide range of published studies [23–25], the findings of this research confirm that extracts of *Allium* species exhibit a significant antibacterial activity.

### 3.4. Antioxidant Activities of Active Compounds in Allium Extracts

This study evaluated the antioxidant activities of active compounds (phenols, allicin, and free amino acids) in *Allium* extracts. *A. fistulosum* extracts showed the highest TPC, followed by *A. sativum*, *A. cepa*, *A. tuberosum*, and *A. fistulosum* L. var. *caespitosum*. For the allicin content, *A. sativum* ranked first, followed by *A. fistulosum*, *A. cepa*, *A. fistulosum* L. var. *caespitosum*, and *A. tuberosum*. In antioxidant capacity assays, *A. fistulosum* extracts exhibited the highest DPPH free radical scavenging rate (69.50%), followed by *A. tuberosum* (54.33%), *A. cepa* (48.63%), *A. sativum* (48.57%), and *A. fistulosum* L. var. *caespitosum* (36.27%). *A. sativum* extracts showed the highest TEAC value (16.30 μmol/L), while *A. tuberosum* extracts demonstrated the strongest SOD antioxidant activity (412.17 U/mg protein), closely followed by *A. fistulosum* (411.83 U/mg protein).

The differences in extraction yields among 19RW, 34PRW, 38KL, and 58KL can be attributed primarily to the effects of solvent polarity and alcohol concentration on the solubility and mass‐transfer behavior of phenolic compounds and allicin. Because the polarity of ethanol–water mixtures decreases with increasing ethanol content, solvents with intermediate alcohol levels (34PRW and 38KL) provide a balanced polarity profile capable of efficiently solubilizing semipolar phenolics and sulfur‐containing compounds. These mixtures maintain sufficient hydrogen‐bonding capacity from water while offering improved organic solubility and enhanced permeability through plant tissues, resulting in superior extraction performance compared with the more polar 19RW or the less polar 58KL. In contrast, the highest alcohol concentration (58KL) reduces the dielectric constant to a level that may limit the dissolution of semipolar constituents and potentially destabilize allicin, whereas the lowest alcohol concentration (19RW) may lack the organic solvency needed for efficient diffusion and release of bioactive molecules. Such biphasic behavior of ethanol–water systems is consistent with solvent extraction theory for phytochemicals. Similar trends have been observed in other *Allium* species, where solvent polarity strongly influences phenolic recovery. Alcohol‐based solvents such as methanol or ethanol typically yield higher TPCs and antioxidant activity than less polar solvents, supporting the superior performance of 34PRW and 38KL in the present study [45].

All alcoholic extracts exhibited notable antioxidant activity, consistent with previous findings that organic sulfur and phenolic compounds in *Allium* species contribute significantly to antioxidant properties [9, 42, 46–49]. Both previous reports [26, 27] and the present study provide evidence that extracts of *Allium* species possess notable antioxidant capacity.

### 3.5. Integrated Discussion: Antioxidant and Antibacterial Activities of Allium Extracts

This study demonstrated that both phenolic compounds and sulfur‐containing metabolites, particularly allicin, play essential roles in the antioxidant and antibacterial properties of *Allium* extracts. Among the species tested, *A. fistulosum* extracts exhibited the highest TPC, whereas *A. sativum* extracts contained the greatest levels of allicin. In antioxidant assays, *A. fistulosum* displayed the strongest DPPH radical scavenging activity (69.50%), while *A. sativum* recorded the highest TEAC value (16.30 μmol/L). Notably, *A. tuberosum* demonstrated superior SOD activity (412.17 U/mg protein), a finding that may be explained by the presence of flavonoids such as quercetin and rutin, as previously reported by Kurnia et al. [42]. These results support the view that both phenolics and sulfur compounds act synergistically in radical scavenging and modulation of endogenous antioxidant enzymes.

Parallel to antioxidant activity, the extracts also exhibited antibacterial effects against major clinical pathogens including *A. baumannii, B. subtilis, E. coli, K. pneumoniae, P. aeruginosa,* and *S. aureus*. *A. sativum* extracts showed the strongest antibacterial activity, consistent with the high allicin content, followed by *A. tuberosum* and *A. fistulosum*. In contrast, *A. cepa* and *A. fistulosum* var. *caespitosum* showed limited activity, inhibiting only specific Gram‐negative bacteria when extracted with 34PRW or 38KL. These findings are in agreement with previous studies highlighting allicin’s broad‐spectrum antibacterial role, especially its ability to target thiol‐containing bacterial enzymes [9, 11, 13, 50].

Taken together, these results indicate that the biological activities of *Allium* extracts are shaped by a balance between their phenolic and sulfur compound profiles. Phenolics provide general antioxidant protection and may enhance SOD activity, while allicin is the principal factor driving antibacterial potency. The use of rice wines and kaoliang liquors as extraction solvents further demonstrates that hydroalcoholic systems efficiently recover both classes of bioactive compounds, underscoring their potential for functional food and nutraceutical applications. The present findings, together with previous reports, confirm that allicin and *Allium* extracts possess pronounced antibacterial activity [23–25], partly through the disruption of bacterial cell membranes. Furthermore, consistent evidence from both this study and the literature demonstrates that *Allium* extracts also exhibit significant antioxidant capacity [26, 27], highlighting their dual functional potential.

Although our correlation analyses suggested distinct contributions of allicin, phenolics, and amino acids to antioxidant and antibacterial activities, a multivariate approach such as principal component analysis (PCA) would provide a more comprehensive visualization of these relationships. Due to the limited dataset in the present study, PCA was not performed; however, we recommend incorporating PCA in future work to better elucidate the integrated effects of these bioactive compounds.

## 4. Conclusion

All extracts demonstrated notable antioxidant activities. *A. fistulosum* had the highest DPPH scavenging rate and phenol content; *A. sativum* showed the highest TEAC value; and *A. tuberosum* exhibited the strongest SOD activity. Moreover, extracts of *A. sativum*, *A. tuberosum*, and *A. fistulosum* displayed effective antibacterial activity against major clinical pathogens. These findings highlight the potential of *Allium* extracts as functional food supplements and alternative food preservatives.

## Disclosure

All authors reviewed and approved the final version of the manuscript.

## Conflicts of Interest

The authors declare no conflicts of interest.

## Author Contributions

Tsan‐Chang Chang was the main supervisor of this research work. Hung‐Der Jang contributed to the study conception, performed the statistical analysis, and performed the experimental steps. All authors contributed to the critical revision of the article.

## Funding

No funding was received for this research.

## Data Availability

The data that support the findings of this study are available from the corresponding author upon reasonable request.
